# Luteal-phase stimulation does not compromise embryo euploidy or live birth outcomes compared to follicular-phase stimulation

**DOI:** 10.3389/fmed.2026.1722250

**Published:** 2026-02-11

**Authors:** Boyuan Chen, Meiling Zhang, Min Wang, Yong Wang, Xiaojun Chen, Li Wang, Wen Li, Dandan Wu

**Affiliations:** 1 The International Peace Maternity and Child Health Hospital, School of Medicine, Shanghai Jiao Tong University, Shanghai, China; 2 Shanghai Key Laboratory of Embryo Original Diseases, Shanghai, China

**Keywords:** assisted reproductive technology, controlled ovarian stimulation, euploidy, luteal-phase stimulation, preimplantation genetic testing for aneuploidy (PGT-A)

## Abstract

**Background:**

Aneuploidy is a major cause of implantation failure and miscarriage in assisted reproductive technology. This study aimed to evaluate embryo euploidy and live birth rates associated with the luteal-phase stimulation (LPS) protocol in *in vitro* fertilization (IVF), compared with the follicular-phase stimulation (FPS) protocol.

**Methods:**

We conducted a retrospective cohort study at a university-affiliated fertility center, including 667 preimplantation genetic testing for aneuploidy (PGT-A) cycles performed between January 2020 and June 2023. After 1:1 propensity score matching, 106 cycles were analyzed from each group (LPS and FPS). Baseline characteristics, ovarian stimulation parameters, euploidy rate, and subsequent reproductive outcomes were compared.

**Results:**

After matching, baseline characteristics were comparable between the groups. The LPS group required a longer duration of gonadotropin stimulation (median 10.5 vs. 9 days, *p* < 0.001) and yielded fewer retrieved oocytes (median 6 vs. 9, *p* < 0.05) than the FPS group. On the day of hCG administration, patients in the LPS group had significantly higher progesterone levels than those in the FPS group (median 6.1 vs. 3.1, *p* < 0.001). However, the euploidy rate did not differ significantly (33.9% vs. 30.8%, *p* > 0.05). Subgroup analyses stratified by age (<38 and ≥38 years) revealed consistent results. Similarly, the clinical pregnancy rate (55.9% vs. 64.1%, *p* > 0.05), live birth rate (41.2% vs. 46.2%, *p* > 0.05), and pregnancy loss rate (14.7% vs. 17.9%, *p* > 0.05) after the first embryo transfer were comparable.

**Conclusion:**

The LPS protocol demonstrated similar embryo euploidy and live birth rates to the FPS protocol, suggesting that LPS does not compromise chromosomal competence or reproductive outcomes. However, the lower oocyte yield in the LPS group could potentially affect cumulative pregnancy and live birth rates. Given its scheduling flexibility, LPS may still represent a clinically feasible and patient-friendly option in assisted reproductive technology (ART).

## Introduction

Controlled ovarian stimulation (COS) is a fundamental component of the IVF cycle. Among the various protocols developed for COS, the gonadotropin-releasing hormone (GnRH) antagonist protocol during the follicular phase is commonly used due to its safety and convenience. However, it poses a challenge for clinicians due to its higher cost (1). Therefore, with the growing application of IVF, there is an increasing need for new approaches that enhance both cost-effectiveness and patient convenience.

The LPS protocol was initially proposed for fertility preservation in 2013 (2). It utilizes negative feedback exerted from elevated progesterone levels during the luteal phase to suppress the luteinizing hormone (LH) surge, while exogenous gonadotropin (Gn) injections are used to increase follicle-stimulating hormone (FSH) levels to obtain sufficient oocytes for IVF. Compared to the conventional FPS protocol, the luteal-phase stimulation (LPS) protocol enables the initiation of ovarian stimulation during the luteal phase rather than the early follicular phase. According to the follicle wave theory, multiple waves of follicle recruitment occur during the human menstrual cycle, with two or three waves possible in each cycle (3). LPS leverages the development of multiple follicles, offering a more flexible approach for individuals requiring urgent fertility preservation or those seeking to optimize their time. Furthermore, LPS, when combined with human menopausal gonadotropin (HMG) and letrozole, significantly reduces the risk of ovarian hyperstimulation syndrome (OHSS) (4).

Numerous studies have investigated the LPS protocol, demonstrating that LPS is non-inferior to conventional protocols in terms of pregnancy outcomes (5). Studies targeting LPS in poor ovarian responders indicate that it offers comparable efficacy and may even enhance ovarian responsiveness (6–8). In addition, LPS is more patient-friendly, with its lower costs and fewer injections, and offers greater flexibility (9, 10). Moreover, children born to patients who underwent the LPS protocol exhibit similar outcomes to those born to patients who underwent conventional protocols, supporting the long-term safety of LPS (11). However, some clinical studies have raised concerns about the potential negative effects exerted from elevated progesterone levels in the LPS protocol on embryos (12, 13).

Aneuploidy is a key factor in both miscarriage and implantation failure (14), accounting for approximately 50% of early pregnancy losses (15). For patients undergoing PGT-A cycles to guide embryo selection, obtaining euploid embryos is a critical objective of ovarian stimulation (OS). To our knowledge, no published data currently exist regarding embryo euploidy rates in patients treated with LPS, despite the strong correlation between euploidy status and the likelihood of achieving a live birth.

This retrospective study aimed to determine whether elevated progesterone levels during the luteal phase influence embryo development and whether the LPS protocol negatively affects embryo ploidy.

## Methods

### Study design and population

This retrospective cohort study included patients who underwent oocyte retrieval with PGT-A between January 2020 and June 2023 at the International Peace Maternity & Child Health Hospital of China Welfare Institute. Only the patient’s first three cycles were included. The study protocol was approved by the ethics committee of the International Peace Maternity & Child Health Hospital.

The criteria for exclusion were as follows: (1) age at retrieval > 45 years, (2) menstrual cycle disorder with antral follicle count ≥ 20 in both ovaries, (3) FSH > 15 IU/L, (4) patients’ indications for preimplantation genetic testing for structural rearrangement or preimplantation genetic testing for monogenic disorders, including those with abnormal parental karyotype or a diagnosis of a monogenic disease, and (5) missing core data.

All cycles were classified into the LPS group and the FPS group, as determined by the clinician based on the patient’s condition. To address the imbalance in baseline characteristics between the two groups, propensity score matching was performed.

### IVF procedures and PGT-A

All women underwent either a luteal-phase stimulation protocol or a GnRH antagonist protocol under the guidance of clinicians. The basal serum levels of follicle-stimulating hormone (FSH) and luteinizing hormone (LH) were measured on menstrual cycle day 2 or 3, as well as the antral follicle count (AFC), which was determined by transvaginal ultrasound. In the FPS group, ovarian stimulation commenced on cycle day 3. Patients received gonadotropins (150–225 IU/day). When at least one follicle reached a mean diameter of 12 mm or more (calculated from the two largest diameters), a daily dose of 0.25 mg GnRH antagonist was initiated and continued until the day of human chorionic gonadotropin (hCG) administration. In the LPS group, ovarian stimulation was initiated during the luteal phase on days 3–5 after ovulation. Patients were administered gonadotropins (150–225 IU/day) and letrozole (Hengrui, Jiangsu, China) (5 mg/day). The gonadotropins used in this study included recombinant follicle-stimulating hormone (rFSH) (Gonal-F, Merck Serono, Italy, or Puregon, Organon, Oss, Netherlands) and human menopausal gonadotropins (HMG; Zhuhai Lizhu Pharmaceutical Co., Ltd.). Follicular development was monitored via transvaginal ultrasound every 1–4 days, beginning 5–6 days after the initiation of ovarian stimulation. These doses were adjusted based on the ovarian response, which was monitored through ultrasonography and serum sex steroid measurements. On the trigger day, hCG (either 250 μg or 6,500 IU) was administered when at least two follicles reached a diameter of 18 mm or greater. Oocyte retrieval was performed under transvaginal ultrasound guidance 36 (±2) h later.

After a period of 2–7 days of abstinence from sexual activity, fresh sperm samples were collected during masturbation. *In vitro* fertilization of the oocytes was performed using an intracytoplasmic sperm injection (ICSI), in which a single sperm was injected into each mature, denuded oocyte. Only metaphase II (MII) oocytes that had extruded the first polar body were selected for microinjection. Fertilization was assessed approximately 16–18 h post-ICSI on day 1. Normal fertilization was confirmed by the presence of a second polar body and the observation of two pronuclei.

On the third day after oocyte retrieval, approximately 68 h post-ICSI or insemination, embryo morphology was evaluated (16). Based on the size of the blastomeres and the degree of anucleate fragmentation, embryos were classified into four distinct morphological grades according to established criteria (17). Good-quality blastocysts were defined as those with normal fertilization and a morphological grade of ≥4BB on day 5 or 6. Blastocyst quality was assessed using the Gardner blastocyst grading system (18).

Embryo biopsy was conducted at the blastocyst stage using laser technology (NARISHIGE, Japan). A small number of trophectoderm cells were withdrawn from each blastocyst, depending on its quality and morphology. The blastocysts selected for biopsy was hatched on the same day as the biopsy procedure. Embryos demonstrating developmental potential based on morphology were biopsied, and only blastocysts of medium quality (3 BC) or higher were selected for subsequent freezing and biopsy. The results of PGT-A were classified as euploid, aneuploid, or mosaic, corresponding to less than 30%, greater than 70, and 30 to 70% of abnormal cells, respectively.

Embryo transfer and luteal support protocols were tailored to each patient’s specific circumstances, as determined by the attending physicians. All embryos in both groups underwent a freeze-all strategy, with no fresh embryo transfers performed. The majority of patients preparing for a frozen embryo transfer (FET) adhered to an artificial regimen to prepare the endometrium, while some patients with regular menstrual cycles adhered to a natural regimen. For those on the artificial regimen, vaginal progesterone gel (Crinone, 90 mg daily or Utrogestan, 600 mg daily) was administered in conjunction with oral dydrogesterone (Duphaston, 10 mg twice daily) and continued until serum hCG testing. Patients who underwent a natural ovulation cycle began treatment with oral dydrogesterone (Duphaston, 10 mg twice daily) on the day of ovulation and continued until serum hCG testing. In each transfer cycle, only one embryo was transferred per patient in both groups. If pregnancy was confirmed, luteal phase support was maintained until 11 weeks of gestation.

### Study outcomes

The primary outcome of the study was the euploidy rate, defined as the ratio of euploid embryos to the total number of embryos biopsied.

Secondary outcomes included the number of oocytes retrieved, the oocyte fertilization rate, the clinical pregnancy rate after the first embryo transfer, and the live birth rate after the first embryo transfer. Specifically, the MII rate was determined by dividing the count of metaphase II oocytes by the count of oocytes retrieved; the fertilization rate was determined by dividing the count of fertilized oocytes by the count of metaphase II oocytes; the 2PN rate was determined by dividing the count of two pronuclei stage zygotes by the count of metaphase II oocytes; the cleavage rate was determined by dividing the count of cleavage stage embryos by the count of fertilized oocytes; the good-quality embryo on D3 rate was determined by dividing the count of good-quality embryos on day 3 by the count of D3 embryos; the blastocyst formation rate was determined by dividing the count of blastocysts by the count of 2PN embryos cultured; the good-quality blastocyst rate was determined by dividing the count of good-quality blastocysts by the count of blastocysts; the questionable rate was determined by dividing the count of embryos with unknown sequencing results due to amplification failure by the total count of embryos biopsied.

Transferable embryos were defined as euploid embryos after biopsy. Biochemical pregnancy was defined as a serum human chorionic gonadotropin level of at least 25 mU per milliliter at 14 days after embryo transfer. Clinical pregnancy was defined as the visualization of the intrauterine gestational sac via ultrasonography approximately 28 days post-transfer. Pregnancy loss was defined as pregnancies that eventuate in a spontaneous abortion or therapeutic abortion that occurred throughout pregnancy. Live birth was defined as the delivery of any viable neonate who was 28 weeks of gestation or older (19). All transfer outcomes were followed up until August 2025.

### Statistical analysis

Continuous variables were assessed for distribution using the Kolmogorov–Smirnov or Shapiro–Wilk test and were reported as means (±SD) and medians (IQR) for non-normally distributed continuous variables. Data with a normal distribution were analyzed using the Student’s *t*-test, whereas the Mann–Whitney U-test was used for non-normally distributed data. The frequencies and percentages of categorical variables were compared using either Pearson’s chi-square test or Fisher’s exact test, as appropriate. Propensity scores were calculated using a logistic regression model that incorporated variables including indications for PGT-A, age, body mass index (BMI), number of prior ovarian stimulation cycles, and anti-Müllerian hormone (AMH) levels. Propensity score matching (PSM) was performed without replacement using the nearest neighbor random matching algorithm, with a caliper width of 0.2 and a match ratio of 1:1. A *p*-value of less than 0.05 was considered statistically significant for all two-tailed tests. The analyses were conducted using Statistical Package for Social Sciences (SPSS) software, version 26.0 (IBM, United States).

## Results

**Figure 1:**
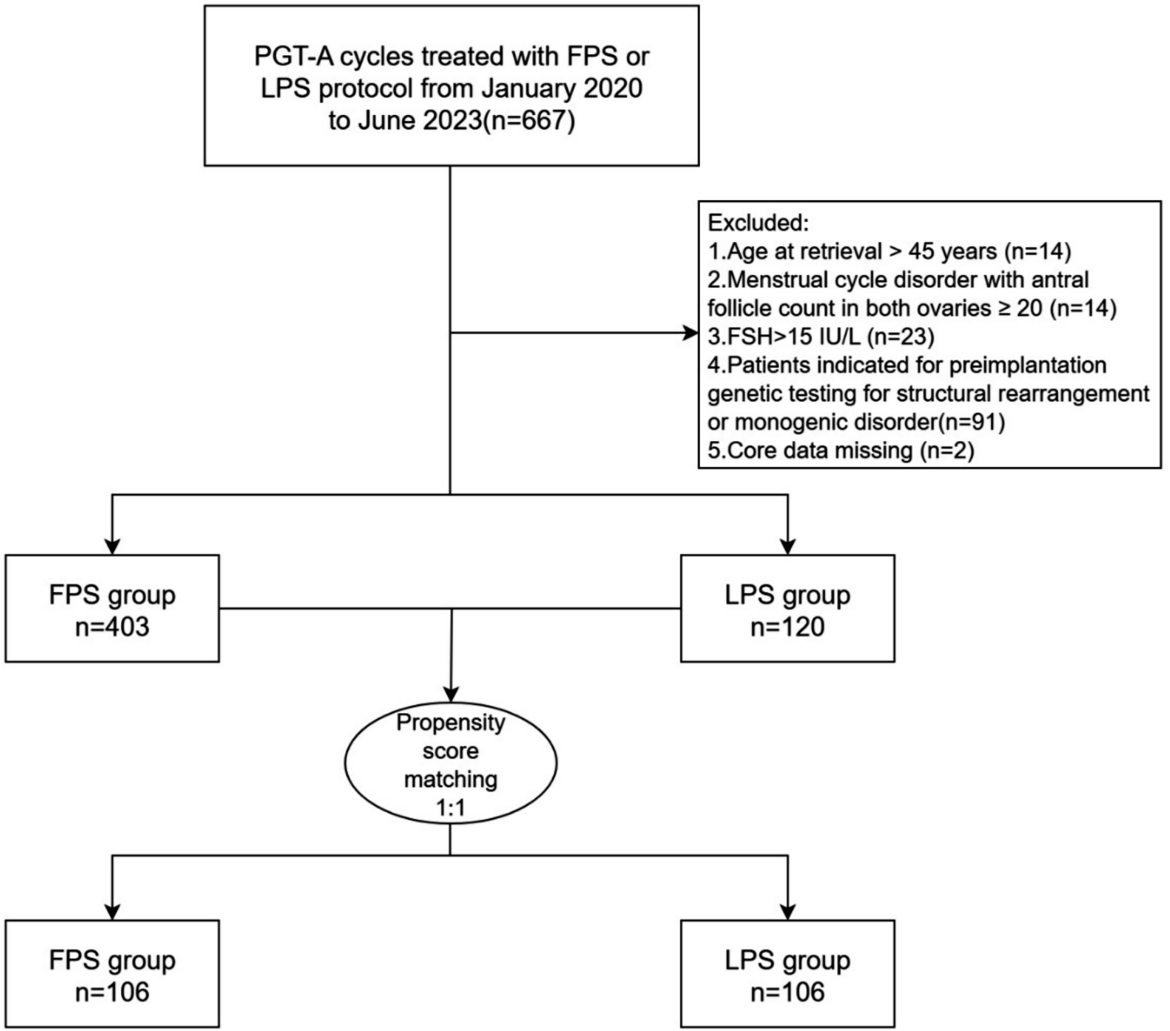
Flowchart of the study population.

Initially, a total of 667 PGT-A cycles that were treated with either the FPS or LPS protocol were screened between January 2020 and June 2023. After screening and propensity score matching, 212 PGT-A cycles were included in this study, with 106 cycles in the LPS group and 106 cycles in the FPS group (Figure 1). Follow-up with all participants was completed in August 2025.

After PSM, the baseline characteristics were found to be highly comparable between the two groups (Table 1). The median maternal age was 39.6 years in the LPS group and 39.8 years in the FPS group. The indications for PGT-A and AMH levels were similar in both groups.

**Table 1:** Baseline characteristics of the LPS and the FPS group after propensity score matching.

Characteristics	LPS group (*n* = 106)	FPS group (*n* = 106)	*P*-value
Maternal age, years	39.6 (36.3–42.3)	39.8 (36.6–41.3)	0.51
Paternal age, years	40.0 (35.7–43.7)	40.5 (26.6–43.4)	0.348
BMI, kg/m^2^	21.8 (19.5–23.5)	22.0 (20.1–23.4)	0.49
Education level, no. (%)			0.152
Below high school	9 (8.5)	4 (3.8)	
High school or above	97 (91.5)	102 (96.2)	
Area, no. (%)			0.68
Shanghai	50 (47.2)	53 (50.0)	
Others	56 (52.8)	53 (50.0)	
Infertility types, no. (%)			0.579
Primary	16 (15.1)	19 (17.9)	
Secondary	90 (84.9)	87 (82.1)	
Infertility causes, no. (%)			0.836
Female factor	85 (80.2)	85 (80.2)	
Male factor	1 (0.9)	2 (1.9)	
Both female and male factors	20 (18.9)	19 (17.9)	
Indications for PGT-A, no. (%)			0.924
Advanced maternal age	24 (22.6)	20 (18.9)	
Recurrent pregnancy loss	56 (52.8)	58 (54.7)	
Recurrent implantation failures	11 (10.4)	12 (11.3)	
Male factors and others	15 (14.2)	16 (15.1)	
Basal FSH, IU/L	7.9 (6.8–9.8)	7.4 (6.6–9.2)	0.209
Basal LH, IU/L	4.0 (2.8–5.5)	4.3 (3.2–5.2)	0.476
AMH, ng/ml	2.0 (0.8–3.1)	2.0 (1.3–3.3)	0.203
AFC	7 (5–11)	8 (6–11)	0.192

Continuous variables are presented as the median (first quartile–third quartile). Categorical variables are presented as No. (%).

Table 2 presents the characteristics and outcomes of ovarian stimulation. Patients in the LPS group were treated with gonadotrophins for a longer duration than those in the FPS group (10.5 (9–12) vs. 9 (8–10), *p* < 0.001). On the day of hCG administration, patients in the LPS group showed notably lower estradiol levels (*p* < 0.001) and higher progesterone levels (*p* < 0.001). Notably, fewer oocytes were retrieved from patients in the LPS group compared to those in the FPS group (6 (3–10) vs. 9 (5–13), *p* = 0.003). The number of MII oocytes in the LPS group was also lower (5 (3–8) vs. 6 (4–11), *p* = 0.011). Consequently, the LPS group had fewer embryos on day 3 (4 (2–7) vs. 5 (3–9), *p* = 0.012) and fewer blastocysts (2 (0–4) vs. 3 (1–5), *p* = 0.026) than the FPS group. The proportion of cycles with at least one blastocyst was 69.8% in the LPS group and 88.1% in the FPS group, with the difference approaching but not reaching statistical significance (*p* = 0.055).

**Table 2:** Characteristics and outcomes of ovarian stimulation in the LPS and the FPS group after matching.

Variables	LPS group (*n* = 106)	FPS group (*n* = 106)	*P*-value
Gn dosage (IU)	2187.5 (156.3–2662.5)	2325.0 (2025.0–2850.0)	0.134
Gn duration (days)	10.5 (9–12)	9 (8–10)	<0.001
LH on the day of hCG administration (IU/L)	2.4 (1.8–4.6)	3.0 (2.1–4.3)	0.36
E2 on the day of hCG administration (pmol/l)	2088.0 (826.5–5882.0)	9412.8 (4708.6–14674.5)	<0.001
P on the day of hCG administration (nmol/l)	6.1 (3.6–18.4)	3.1 (2.2–4.4)	<0.001
No. of oocytes retrieved	6 (3–10)	9 (5–13)	0.003
No. of MII oocytes	5 (3–8)	6 (4–11)	0.011
No. of embryos on D3	4 (2–7)	5 (3–9)	0.012
No. of blastocysts	2 (0–4)	3 (1–5)	0.026
No. of good-quality blastocysts	0 (0–1)	1 (0–1)	0.169
The percentage of cycles with ≥ 1 blastocyst, no./total no. (%)	74/106 (69.8)	86/106 (88.1)	0.055
No. of blastocysts biopsied	2 (1–3)	2 (1–4)	0.053

Continuous variables are presented as the median (first quartile–third quartile).

Laboratory outcomes are presented in Table 3. Although fewer oocytes were retrieved in the LPS group, the MII rate, fertilization rate, 2PN rate, cleavage rate, good-quality embryo on day 3 rate, good-quality blastocyst rate, and blastocyst formation rate were similar between the two groups. The euploidy rate was also comparable between the two groups (33.9% vs. 30.8%, *p* = 0.452), as were the rates of mosaicism and aneuploidy. In the LPS group, five embryos (2.2%) were classified as questionable, compared to two embryos (0.7%) in the FPS group. Subgroup analysis of patients aged <38 years and ≥38 years revealed no difference in the euploidy rate between the two groups (Table 4).

**Table 3:** Laboratory outcomes of the LPS and FPS groups after matching.

Variables	LPS group (*n* = 106)	FPS group (*n* = 106)	*P*-value
MII rate, no./total no. (%)	624/778 (80.2)	788/984 (80.1)	0.948
Fertilization rate, no./total no. (%)	551/624 (88.3)	697/788 (88.5)	0.93
2PN rate, no./total no. (%)	497/624 (79.6)	625/788 (79.3)	0.878
Cleavage rate, no./total no. (%)	539/551 (97.8)	679/697 (97.4)	0.643
Good-quality embryo on D3 rate, no./total no. (%)	279/530 (52.6)	322/665 (48.4)	0.147
Blastocyst formation rate, no./total no. (%)	255/497 (51.3)	328/625 (52.5)	0.696
Good-quality blastocyst rate, no./total no. (%)	78/255 (34.7)	97/328 (29.6)	0.791
Euploidy rate, no./total no. (%)	78/230 (33.9)	84/273 (30.8)	0.452
Mosaicism rate, no./total no. (%)	41/230 (17.8)	48/273 (17.6)	0.943
Aneuploidy rate, no./total no. (%)	106/230 (46.1)	139/273 (50.9)	0.280
Questionable rate, no./total no. (%)	5/230 (2.2)	2/273 (0.7)	0.321

**Table 4:** Subgroup analysis of euploid embryos between the LPS and FPS groups after matching.

Variables	LPS group (*n* = 106)	FPS group (*n* = 106)	*P*-value
Euploidy rate, no./total no. (%)			
<38 years	46/95 (48.4)	48/118 (40.7)	0.258
≥38 years	32/135 (23.7)	36/155 (23.2)	0.924
Mosaicism rate, no./total no. (%)			
<38 years	23/95 (24.2)	24/118 (20.3)	0.498
≥38 years	18/135 (13.3)	24/155 (15.5)	0.604
Aneuploidy rate, no./total no. (%)			
<38 years	24/95 (25.2)	44/118 (37.3)	0.061
≥38 years	82/135 (60.7)	95/155 (61.3)	0.924

There were 43 cycles with transferable embryos in the LPS group and 45 cycles in the FPS group (Table 5). Of these, embryo transfer was performed in 34 out of 43 cycles (79.1%) in the LPS group and 39 out of 45 cycles (86.7%) in the FPS group (*p* = 0.343). Embryo transfer was not performed for certain patients due to the limited follow-up time. The percentage of good-quality embryos and the percentage of patients undergoing an artificial cycle were similar between the two groups. Clinical pregnancy after the first embryo transfer was observed in 19 (55.9%) cycles in the LPS group and 25 (64.1%) cycles in the FPS group (*p* = 0.474). The data revealed that the biochemical pregnancy rate, pregnancy loss rate, live birth rate, preterm birth rate, and ectopic pregnancy rate after the first embryo transfer were not significantly different between the two groups. Additionally, no cases of twin pregnancy were observed in the study.

**Table 5:** Clinical outcomes after the first embryo transfer of the LPS and FPS groups after matching.

Variables	LPS group (*n* = 106)	FPS group (*n* = 106)	*P*-value
No. of cycles with transferable embryos	43	45	/
Cycles in which patients had embryos transferred, no./total no. (%)	34/43 (79.1)	39/45 (86.7)	0.343
The percentage of good-quality blastocysts transferred	17/34 (50)	19/39 (48.7)	0.913
The percentage of artificial cycle FET	27/34 (79.4)	32/39 (82.1)	0.775
Biochemical pregnancy rate after first embryo transfer, no./total no. (%)	23/34 (67.6)	31/39 (79.5)	0.250
Clinical pregnancy rate after the first embryo transfer, no./total no. (%)	19/34 (55.9)	25/39 (64.1)	0.474
Pregnancy loss rate after the first embryo transfer, no./total no. (%)	5/34 (14.7)	7/39 (17.9)	0.709
Live birth rate after the first embryo transfer^*^, no./total no. (%)	14/34 (41.2)	18/39 (46.2)	0.669
Preterm birth rate after the first embryo transfer, no./total no. (%)	0/34 (0)	3/39 (7.7)	0.243
Ectopic pregnancy rate after the first embryo transfer, no./total no. (%)	1/34 (2.9)	0/39 (0)	0.466

## Discussion

In our study, we found that, although fewer oocytes were retrieved from patients in the LPS group, the euploidy rates, along with clinical pregnancy rates, pregnancy loss rates, and live birth rates after the first embryo transfer, were comparable to those in the FPS group. These findings suggest that the LPS protocol is non-inferior to the FPS protocol in terms of embryo quality and clinical outcomes. Our data may provide new insights into the impact of the LPS protocol on embryo quality and could assist clinicians in their practice.

Our data revealed that patients treated with the LPS protocol had elevated progesterone levels on the day of hCG administration. This elevation can be attributed to the luteal phase, during which follicles are expelled to form the corpus luteum, which secretes increased progesterone, thereby creating a high progesterone state that effectively controls the LH surge. Similarly, progestin-primed ovarian stimulation (PPOS) also facilitates better control of LH concentrations due to the administration of exogenous progestin (20).

Some clinical studies have raised concerns about the potential adverse effects of high serum progesterone levels on embryo quality (12, 13). Recent studies have recommended that protocols using medroxyprogesterone acetate (MPA) and conventional protocols, such as the short protocol, result in similar numbers of mature oocytes retrieving and high-quality embryos (21–23). However, despite comparable clinical outcomes between the PPOS protocol and traditional protocols, lower euploidy and blastocyst formation rates have been observed in older patients using the PPOS protocol (24). Another study has found that the rate of formation of euploid blastocysts per oocyte was comparable between patients undergoing PPOS and those following the conventional protocol (25). Our results supported the latter finding, indicating that elevated progesterone levels did not lead to a reduced euploidy rate.

The conventional GnRH antagonist protocol did not include letrozole, which was added to the LPS protocol, leading to differences in estrogen levels on the trigger day. Our study indicated that low estrogen levels following letrozole administration did not affect oocyte maturation or embryo development, as discussed previously (26). Notably, our study is distinctive owing to the fact that prior research did not focus on the impact of LPS on the euploidy rate, a critical factor in successful IVF outcomes.

Proposed more than a decade ago, the LPS protocol was initially not widely adopted due to its inefficacy, high gonadotropin consumption, and relatively prolonged stimulation period (2). To explore its potential for routine use in IVF, independent of menstrual cycles, Kuang Y et al. showed that hMG and letrozole could initiate ovarian stimulation during the luteal phase. This approach successfully produced competent oocytes and embryos, leading to optimal pregnancy outcomes while also reducing gonadotropin doses and stimulation days (4). In a large retrospective cohort study, they further reported that this novel LPS strategy achieved outcomes, including mature oocytes retrieved and top-quality embryos obtained, comparable to conventional mild treatment and short-term protocols (27). Additionally, a study comparing PPOS, the GnRH antagonist protocol, and LPS for fertility preservation found no significant difference in the number of oocytes retrieved among the three groups (10). However, our study showed that fewer oocytes were retrieved from the LPS group. Several potential explanations may account for this observation. First, LPS involves higher gonadotropin doses over a longer duration compared to FPS, indicating that elevated progesterone levels during the luteal phase may inhibit follicle development, leading to a reduced oocyte yield (12). Second, AFC was measured during the early follicular phase (days 2–3) of the menstrual cycle, but AFC during the luteal phase was not assessed. It is possible that the AFC in the luteal phase, when LPS is initiated, is lower than that in the follicular phase, further contributing to the observed differences in oocyte retrieval.

Nevertheless, our results indicated that the LPS protocol demonstrated comparable clinical pregnancy rates, pregnancy loss rates, and live birth rates to the FPS protocol, consistent with findings from other studies. A meta-analysis of 12 studies concluded that clinical pregnancy rates and live birth rates for patients receiving LPS are comparable to those who initiated ovarian stimulation during the follicular phase (5). A multicenter study also reported that miscarriage rates and live birth rates following blastocyst transfer from LPS-derived embryos were comparable to those from FPS (28). Although there was no statistically significant difference in pregnancy outcomes between the LPS and FPS groups, the LPS group showed a slight disadvantage. Additionally, while there were no statistically significant differences, the patients undergoing embryo transfer in the LPS group had slightly higher age (38.3 (34.6–40.0) vs. 37.5 (34.2–39.0), *p* = 0.276) and BMI (22.15 (19.92–24.05) vs. 21.83 (19.56–22.64), *p* = 0.765) compared to the FPS group. These differences, although not statistically significant, may contribute to the observed trend of inferior clinical outcomes in the LPS group. Furthermore, elevated progesterone levels during the luteal phase may negatively impact oocyte cytoplasmic maturation and embryo development, potentially influencing overall pregnancy outcomes (13).

Our study has several limitations. First, being observational and retrospective introduces the potential for selection bias, as there was no randomization between groups. Additionally, this study was conducted at a single site with a limited sample size, which may limit the generalizability of our findings. Another limitation is the insufficient follow-up time to assess cumulative clinical pregnancy rates; consequently, we reported only the clinical pregnancy rate after the first embryo transfer as our study outcome. Furthermore, the lower oocyte yield observed in the LPS group may have implications for cumulative pregnancy and live birth rates, which should be further explored in future studies with larger sample sizes and longer follow-up. Overall, more reliable results would require a randomized controlled trial.

## Conclusion

In summary, our findings suggest that the LPS and FPS protocols yield comparable euploidy rates and pregnancy outcomes. There was no evidence indicating that elevated progesterone levels in the LPS protocol negatively affected embryo euploidy status. Compared to the FPS protocol, LPS offers greater flexibility in the timing of ovulation stimulation initiation and holds promise for broader clinical applications. The effectiveness and safety of the LPS protocol could be more clearly evaluated in a randomized controlled trial.

## Data Availability

The data analyzed in this study is subject to the following licenses/restrictions: data will be made available for review or query upon reasonable request. Requests to access these datasets should be directed to DW, woodendenny@sjtu.edu.cn.

## Glossary

PGT-A: preimplantation genetic testing for aneuploidy
LPS: luteal-phase stimulation
FPS: follicular-phase stimulation
COS: controlled ovarian stimulation
IVF: *in vitro* fertilization
ART: assisted reproductive technology
GnRH: gonadotropin-releasing hormone
Gn: gonadotropin
HMG: human menopausal gonadotropin
OHSS: ovarian hyperstimulation syndrome
OS: ovarian stimulation
hCG: human chorionic gonadotropin
ICSI: intracytoplasmic sperm injection
NGS: next-generation sequencing
PSM: propensity score matching
BMI: body mass index
FSH: follicle stimulation hormone
LH: luteinizing hormone
AMH: anti-Müllerian hormone
AFC: antral follicle counting
E2: estradiol
P: progesterone
MII: metaphase II
2PN: two pronuclei
PPOS: progestin-primed ovarian stimulation
MPA: medroxyprogesterone acetate
